# Bis[μ-1,2-bis­(1*H*-imidazol-1-ylmethyl)benzene-κ^2^ *N* ^3^:*N* ^3′^]disilver(I) bis­(4-amino-2,5-dichloro­benzene­sulfonate) tetra­hydrate

**DOI:** 10.1107/S1600536808023052

**Published:** 2008-07-26

**Authors:** Hai-Yan Liu, Yun-Chao Chi, Guang-Hui Wang

**Affiliations:** a Department of Chemistry and Pharmaceutical Engineering, Suihua University, Suihua 152061, People’s Republic of China

## Abstract

The asymmetric unit of the title compound, [Ag_2_(C_14_H_14_N_4_)_2_](C_6_H_4_Cl_2_NO_3_S)_2_·4H_2_O, contains one-half of each of two independent dicationic units, two 4-amino-2,5-dichloro­benzene­sulfonate anions and four water mol­ecules. Each centrosymmetric dicationic unit has a dinuclear structure in which two Ag^I^ atoms are bridged by two 1,2-bis­(1*H*-imidazol-1-yl­meth­yl)benzene ligands in a slightly distorted linear coordination geometry. The 4-amino-2,5-dichloro­benzene­sulfonate anion does not coordinate with the Ag^I^ center, acting only as a counteranion. In the crystal structure, inter­molecular O—H⋯O and N—H⋯O hydrogen bonds form a three-dimensional network.

## Related literature

For related literature, see: Aakeröy & Beatty (1998 ▶); Cote & Shimizu (2004 ▶); Feazell *et al.* (2006 ▶); Li *et al.* (2006 ▶); Liu *et al.* (2007 ▶); Ma *et al.* (2005 ▶).
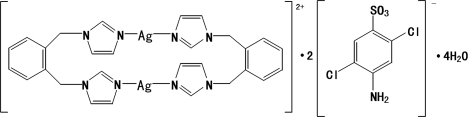


## Experimental

### 

#### Crystal data

[Ag_2_(C_14_H_14_N_4_)_2_](C_6_H_4_Cl_2_NO_3_S)_2_·4H_2_O
*M* *_r_* = 1246.51
Triclinic, 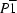

*a* = 11.732 (6) Å
*b* = 14.598 (6) Å
*c* = 15.718 (6) Å
α = 79.068 (12)°
β = 72.843 (19)°
γ = 70.991 (17)°
*V* = 2418.8 (18) Å^3^
*Z* = 2
Mo *K*α radiation
μ = 1.18 mm^−1^
*T* = 293 (2) K
0.35 × 0.25 × 0.24 mm

#### Data collection

Rigaku R-AXIS RAPID diffractometer
Absorption correction: multi-scan (*ABSCOR*; Higashi, 1995 ▶) *T*_min_ = 0.716, *T*_max_ = 0.753
19762 measured reflections
10576 independent reflections
6897 reflections with *I* > 2σ(*I*)
*R*_int_ = 0.022

#### Refinement

*R*[*F* ^2^ > 2σ(*F* ^2^)] = 0.044
*wR*(*F* ^2^) = 0.152
*S* = 0.96
10576 reflections
649 parameters
13 restraints
H atoms treated by a mixture of independent and constrained refinement
Δρ_max_ = 0.92 e Å^−3^
Δρ_min_ = −0.36 e Å^−3^

### 

Data collection: *PROCESS-AUTO* (Rigaku, 1998 ▶); cell refinement: *PROCESS-AUTO*; data reduction: *PROCESS-AUTO*; program(s) used to solve structure: *SHELXS97* (Sheldrick, 2008 ▶); program(s) used to refine structure: *SHELXL97* (Sheldrick, 2008 ▶); molecular graphics: *SHELXTL-Plus* (Sheldrick, 2008 ▶); software used to prepare material for publication: *SHELXL97*.

## Supplementary Material

Crystal structure: contains datablocks global, I. DOI: 10.1107/S1600536808023052/ci2638sup1.cif

Structure factors: contains datablocks I. DOI: 10.1107/S1600536808023052/ci2638Isup2.hkl

Additional supplementary materials:  crystallographic information; 3D view; checkCIF report

## Figures and Tables

**Table:** 

N2—Ag2	2.092 (3)
N3—Ag2	2.090 (4)
N8—Ag1	2.103 (3)
N9—Ag1	2.100 (3)

**Table:** 

N9—Ag1—N8	175.91 (13)
N3—Ag2—N2	178.95 (15)

**Table 2:** Hydrogen-bond geometry (Å, °)

*D*—H⋯*A*	*D*—H	H⋯*A*	*D*⋯*A*	*D*—H⋯*A*
O2*W*—H2*A*⋯O6	0.81 (6)	2.15 (7)	2.868 (5)	148 (7)
O3*W*—H3*A*⋯O2	0.82 (7)	2.00 (7)	2.819 (6)	172 (9)
O1*W*—H1*A*⋯O4^i^	0.80 (6)	1.99 (6)	2.762 (5)	165 (7)
N6—H6*B*⋯O2^ii^	0.80 (3)	2.19 (4)	2.928 (5)	154 (6)
N5—H5*B*⋯O5^iii^	0.81 (6)	2.28 (6)	2.913 (5)	136 (5)

Symmetry codes: (i) 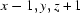; (ii) 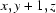; (iii) 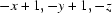.

**Fig. 1.**
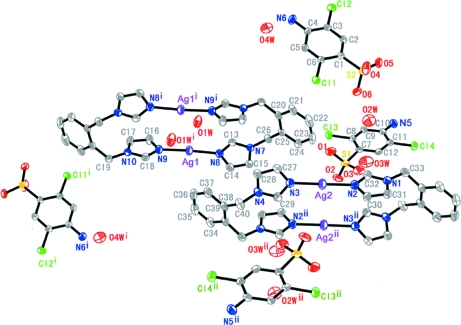
The structure of the title compound, showing the atomic numbering scheme. Displacement ellipsoids are drawn at the 30% probability level. Symmetry code: (i) 1-x, 1-y, 2-z; (ii) 2-x, -y, 1-z.

## supplementary crystallographic information

### Comment

Ag^I^ complexes have shown versatility of their coordination geometry (Aakeröy & Beatty, 1998; Ma *et al.*, 2005). Some silver(I) sulfonate compounds, modified by secondary nitrogen-based ligands, have been reported (Cote & Shimizu, 2004; Liu *et al.*, 2007). Herein, we present a new silver-sulfonate complex, namely [Ag_2_(IBI)_2_]*L*_2_.4H_2_O, where IBI is 1,2-bis ((1*H*-imidazol-1-yl)methyl)benzene and *L* is 4-amino-2,5-dichlorobenzenesulfonic acid.

Selected bond distances and angles are listed in Table 1. The asymmetric unit of the title compound contains one-half each of two independent dicationic units, two 4-amino-2,5-dichlorobenzenesulfonate anions and four water molecules. Each Ag^I^ ion is two-coordinated by two N atoms from two IBI ligands, showing a slightly distorted linear geometry. The Ag—N bond distances are within the normal range observed in N-containing Ag^I^ complexes (Li *et al.*, 2006; Feazell *et al.*, 2006). The *L* anion does not coordinate with silver ion but acts as a counteranion.

N—H···O and O—H···O hydrogen bonds between water molecules and *L* ligands result in the formation of a three-dimensional supramolecular structure (Table  2).

### Experimental

An aqueous solution (10 ml) of 4-amino-2,5-dichlorobenzenesulfonic acid (1 mmol) was added to solid Ag_2_CO_3_ (0.5 mmol) and stirred for several minutes until no further CO_2_ was given off. 1-(3-(1*H*-Imidazol-1-yl)methyl)benzyl)-1*H*-imidazole (1 mmol) was then added and a precipitate was formed. The precipitate was dissolved by ammonium hydroxide. Single crystals of the title compound were obtained by slow evaporation of the solution for 6 d at room temperature.

### Refinement

H atoms bonded to N atoms were located in a difference map and refined with a N—H distance restraint of 0.85 (3) Å and with *U*_iso_(H) = 1.5*U*_eq_(N). Water H atoms were located in a difference Fourier map and refined with O—H and H···H distance restraints of 0.85 (3) Å and 1.30 (3) Å, respectively, and with *U*_iso_(H) = 1.5*U*_eq_(O). H atoms bonded to C atoms were positioned geometrically (C—H = 0.93 or 0.97 Å) and refined as riding, with *U*_iso_(H) = 1.2*U*_eq_(C). The highest residual density peak is located 0.89 Å from atom Ag2 and the deepest hole is located 1.54 Å from atom Ag1.

### Crystal data

**Table:**

[Ag_2_(C_14_H_14_N_4_)_2_](C_6_H_4_Cl_2_N_1_O_3_S)_2_·4H_2_O	*Z* = 2
*M**_r_* = 1246.51	*F*_000_ = 1256
Triclinic, *P*1	*D*_x_ = 1.712 Mg m^−^^3^
Hall symbol: -P 1	Mo *K*α radiation λ = 0.71069 Å
*a* = 11.732 (6) Å	Cell parameters from 10576 reflections
*b* = 14.598 (6) Å	θ = 3.0–27.5º
*c* = 15.718 (6) Å	µ = 1.18 mm^−^^1^
α = 79.068 (12)º	*T* = 293 (2) K
β = 72.843 (19)º	Block, colourless
γ = 70.991 (17)º	0.35 × 0.25 × 0.24 mm
*V* = 2418.8 (18) Å^3^	

### Data collection

**Table:**

Rigaku R-AXIS RAPID diffractometer	10576 independent reflections
Radiation source: fine-focus sealed tube	6897 reflections with *I* > 2σ(*I*)
Monochromator: graphite	*R*_int_ = 0.023
Detector resolution: 10.0 pixels mm^-1^	θ_max_ = 27.5º
*T* = 293(2) K	θ_min_ = 3.0º
ω scans	*h* = −15→15
Absorption correction: multi-scan(ABSCOR; Higashi, 1995)	*k* = −18→18
*T*_min_ = 0.716, *T*_max_ = 0.753	*l* = −20→19
19762 measured reflections	

### Refinement

**Table:**

Refinement on *F*^2^	Secondary atom site location: difference Fourier map
Least-squares matrix: full	Hydrogen site location: inferred from neighbouring sites
*R*[*F*^2^ > 2σ(*F*^2^)] = 0.044	H atoms treated by a mixture of independent and constrained refinement
*wR*(*F*^2^) = 0.152	*w* = 1/[σ^2^(*F*_o_^2^) + (0.0975*P*)^2^] where *P* = (*F*_o_^2^ + 2*F*_c_^2^)/3
*S* = 0.96	(Δ/σ)_max_ = 0.002
10576 reflections	Δρ_max_ = 0.92 e Å^−^^3^
649 parameters	Δρ_min_ = −0.36 e Å^−^^3^
13 restraints	Extinction correction: none
Primary atom site location: structure-invariant direct methods	

### Special details

**Table:**

Geometry. All e.s.d.'s (except the e.s.d. in the dihedral angle between two l.s. planes) are estimated using the full covariance matrix. The cell e.s.d.'s are taken into account individually in the estimation of e.s.d.'s in distances, angles and torsion angles; correlations between e.s.d.'s in cell parameters are only used when they are defined by crystal symmetry. An approximate (isotropic) treatment of cell e.s.d.'s is used for estimating e.s.d.'s involving l.s. planes.
Refinement. Refinement of *F*^2^ against ALL reflections. The weighted *R*-factor *wR* and goodness of fit *S* are based on *F*^2^, conventional *R*-factors *R* are based on *F*, with *F* set to zero for negative *F*^2^. The threshold expression of *F*^2^ > σ(*F*^2^) is used only for calculating *R*-factors(gt) *etc*. and is not relevant to the choice of reflections for refinement. *R*-factors based on *F*^2^ are statistically about twice as large as those based on *F*, and *R*- factors based on ALL data will be even larger.

### Fractional atomic coordinates and isotropic or equivalent isotropic displacement parameters (Å^2^)

**Table:**

	*x*	*y*	*z*	*U*_iso_*/*U*_eq_	
C1	0.8198 (3)	0.6791 (2)	0.2186 (2)	0.0354 (7)	
C2	0.7330 (3)	0.7577 (2)	0.1876 (2)	0.0366 (7)	
H2	0.7202	0.7590	0.1317	0.044*	
C3	0.6652 (3)	0.8340 (2)	0.2383 (2)	0.0388 (7)	
C4	0.6792 (4)	0.8342 (2)	0.3238 (2)	0.0438 (8)	
C5	0.7666 (3)	0.7540 (2)	0.3548 (2)	0.0427 (8)	
H5	0.7784	0.7516	0.4113	0.051*	
C6	0.8349 (3)	0.6793 (2)	0.3037 (2)	0.0356 (7)	
C7	0.3133 (4)	0.2224 (2)	0.3052 (2)	0.0443 (8)	
C8	0.1970 (4)	0.2875 (2)	0.3230 (2)	0.0475 (9)	
C9	0.1282 (4)	0.3219 (3)	0.2605 (2)	0.0480 (9)	
H9	0.0505	0.3674	0.2751	0.058*	
C10	0.1729 (4)	0.2898 (3)	0.1759 (2)	0.0474 (9)	
C11	0.2901 (4)	0.2236 (2)	0.1576 (2)	0.0470 (9)	
C12	0.3595 (4)	0.1902 (2)	0.2207 (3)	0.0458 (8)	
H12	0.4380	0.1457	0.2062	0.055*	
C13	0.4409 (4)	0.3953 (3)	0.8315 (3)	0.0484 (9)	
H13	0.3734	0.4511	0.8355	0.058*	
C14	0.5857 (4)	0.2737 (3)	0.8637 (3)	0.0601 (11)	
H14	0.6393	0.2289	0.8953	0.072*	
C15	0.5879 (4)	0.2697 (3)	0.7782 (3)	0.0538 (10)	
H15	0.6413	0.2227	0.7406	0.065*	
C16	0.2850 (4)	0.5007 (3)	1.1974 (3)	0.0592 (11)	
H16	0.2168	0.5300	1.1731	0.071*	
C17	0.2902 (4)	0.5130 (3)	1.2789 (3)	0.0562 (10)	
H17	0.2282	0.5513	1.3207	0.067*	
C18	0.4640 (4)	0.4143 (3)	1.2125 (3)	0.0525 (9)	
H18	0.5443	0.3719	1.2019	0.063*	
C19	0.4582 (5)	0.4438 (3)	1.3650 (3)	0.0597 (12)	
H19A	0.5467	0.4372	1.3440	0.072*	
H19B	0.4481	0.3839	1.4012	0.072*	
C20	0.6035 (4)	0.4727 (3)	0.5778 (2)	0.0473 (9)	
C21	0.7006 (5)	0.4807 (4)	0.5034 (3)	0.0672 (13)	
H21	0.7272	0.5363	0.4912	0.081*	
C22	0.7575 (5)	0.4091 (5)	0.4482 (3)	0.0814 (17)	
H22	0.8223	0.4163	0.3988	0.098*	
C23	0.7203 (5)	0.3264 (5)	0.4645 (3)	0.0805 (16)	
H23	0.7600	0.2772	0.4268	0.097*	
C24	0.6223 (5)	0.3165 (3)	0.5383 (3)	0.0597 (11)	
H24	0.5958	0.2609	0.5494	0.072*	
C25	0.5645 (4)	0.3890 (3)	0.5949 (2)	0.0426 (8)	
C26	0.4588 (4)	0.3750 (3)	0.6729 (2)	0.0513 (9)	
H26A	0.3906	0.4347	0.6767	0.062*	
H26B	0.4293	0.3241	0.6629	0.062*	
C27	0.9272 (5)	0.2163 (3)	0.6345 (3)	0.0624 (11)	
H27	0.8772	0.2668	0.6042	0.075*	
C28	0.9248 (4)	0.2116 (3)	0.7213 (3)	0.0602 (11)	
H28	0.8742	0.2564	0.7616	0.072*	
C29	1.0624 (4)	0.0856 (3)	0.6622 (3)	0.0555 (10)	
H29	1.1252	0.0270	0.6561	0.067*	
C30	1.2267 (4)	−0.0007 (3)	0.2968 (3)	0.0548 (10)	
H30	1.2907	−0.0338	0.3246	0.066*	
C31	1.2282 (4)	−0.0092 (3)	0.2127 (3)	0.0529 (9)	
H31	1.2920	−0.0478	0.1721	0.064*	
C32	1.0534 (4)	0.0933 (3)	0.2743 (3)	0.0556 (10)	
H32	0.9746	0.1379	0.2823	0.067*	
C33	1.0740 (5)	0.0685 (3)	0.1172 (3)	0.0587 (11)	
H33A	1.1058	0.1188	0.0771	0.070*	
H33B	0.9837	0.0920	0.1323	0.070*	
C34	0.8843 (4)	0.0216 (3)	0.9298 (2)	0.0479 (9)	
C35	0.7849 (5)	0.0306 (4)	1.0058 (3)	0.0628 (12)	
H35	0.7457	−0.0184	1.0247	0.075*	
C36	0.7438 (5)	0.1089 (4)	1.0529 (3)	0.0759 (14)	
H36	0.6781	0.1127	1.1039	0.091*	
C37	0.7994 (6)	0.1817 (4)	1.0251 (3)	0.0802 (15)	
H37	0.7705	0.2360	1.0567	0.096*	
C38	0.8985 (5)	0.1757 (4)	0.9501 (3)	0.0682 (13)	
H38	0.9360	0.2257	0.9318	0.082*	
C39	0.9421 (4)	0.0953 (3)	0.9020 (2)	0.0525 (10)	
C40	1.0505 (4)	0.0916 (4)	0.8221 (3)	0.0634 (12)	
H40A	1.0991	0.1308	0.8293	0.076*	
H40B	1.1034	0.0249	0.8190	0.076*	
N1	1.1171 (3)	0.0505 (2)	0.1992 (2)	0.0470 (7)	
N2	1.1180 (4)	0.0633 (3)	0.3353 (2)	0.0544 (8)	
N3	1.0130 (4)	0.1370 (3)	0.5975 (2)	0.0584 (9)	
N4	1.0121 (3)	0.1272 (2)	0.7374 (2)	0.0509 (8)	
N5	0.1005 (4)	0.3204 (3)	0.1159 (3)	0.0668 (11)	
H5A	0.041 (4)	0.371 (3)	0.130 (4)	0.100*	
H5B	0.140 (6)	0.309 (4)	0.066 (4)	0.100*	
N6	0.6122 (5)	0.9084 (3)	0.3745 (3)	0.0742 (13)	
H6A	0.622 (6)	0.912 (4)	0.424 (3)	0.111*	
H6B	0.580 (6)	0.960 (3)	0.351 (4)	0.111*	
N7	0.4956 (3)	0.3487 (2)	0.75842 (19)	0.0425 (7)	
N8	0.4938 (3)	0.3527 (2)	0.8973 (2)	0.0516 (8)	
N9	0.3948 (3)	0.4387 (2)	1.1558 (2)	0.0516 (8)	
N10	0.4063 (3)	0.4572 (2)	1.2871 (2)	0.0457 (7)	
O1	0.4075 (4)	0.2441 (2)	0.4284 (2)	0.0799 (11)	
O2	0.5249 (3)	0.1193 (2)	0.3297 (2)	0.0792 (10)	
O1W	0.2170 (4)	0.5412 (3)	0.9721 (2)	0.0832 (11)	
H1A	0.169 (7)	0.563 (5)	1.016 (4)	0.125*	
H1B	0.178 (6)	0.515 (4)	0.953 (4)	0.125*	
O3	0.3444 (3)	0.1006 (2)	0.4441 (2)	0.0727 (10)	
O2W	0.7688 (5)	0.3506 (4)	0.1994 (4)	0.1072 (15)	
H2A	0.810 (8)	0.375 (6)	0.216 (5)	0.161*	
H2B	0.794 (8)	0.356 (6)	0.144 (2)	0.161*	
O4	1.0322 (3)	0.5854 (3)	0.1273 (2)	0.0742 (9)	
O3W	0.7592 (4)	0.1554 (4)	0.2732 (4)	0.1118 (16)	
H3A	0.691 (5)	0.145 (5)	0.285 (6)	0.168*	
H3B	0.746 (7)	0.214 (3)	0.254 (5)	0.168*	
O5	0.8507 (3)	0.6085 (2)	0.07362 (17)	0.0578 (7)	
O4W	0.2819 (6)	0.9250 (4)	0.5166 (4)	0.1301 (19)	
H4A	0.299 (8)	0.963 (6)	0.542 (6)	0.195*	
H4B	0.354 (5)	0.902 (7)	0.480 (5)	0.195*	
O6	0.8852 (3)	0.49480 (18)	0.2017 (2)	0.0645 (8)	
S1	0.40489 (11)	0.16866 (7)	0.38332 (8)	0.0534 (3)	
S2	0.90401 (9)	0.58453 (7)	0.15024 (6)	0.0444 (2)	
Ag1	0.44041 (4)	0.40061 (3)	1.02509 (2)	0.06083 (13)	
Ag2	1.06711 (4)	0.09945 (3)	0.46655 (2)	0.06964 (14)	
Cl1	0.94358 (9)	0.58423 (6)	0.34728 (6)	0.0462 (2)	
Cl2	0.55802 (10)	0.93124 (7)	0.19615 (7)	0.0550 (3)	
Cl3	0.12837 (12)	0.33250 (8)	0.42796 (7)	0.0687 (3)	
Cl4	0.35254 (12)	0.17913 (8)	0.05280 (7)	0.0663 (3)	

### Atomic displacement parameters (Å^2^)

**Table:**

	*U*^11^	*U*^22^	*U*^33^	*U*^12^	*U*^13^	*U*^23^
C1	0.0315 (18)	0.0399 (16)	0.0357 (15)	−0.0105 (14)	−0.0085 (13)	−0.0052 (14)
C2	0.0325 (18)	0.0433 (17)	0.0334 (15)	−0.0118 (14)	−0.0083 (13)	−0.0006 (14)
C3	0.0345 (19)	0.0360 (16)	0.0422 (17)	−0.0089 (14)	−0.0091 (14)	0.0014 (15)
C4	0.049 (2)	0.0370 (16)	0.0439 (18)	−0.0071 (15)	−0.0124 (16)	−0.0087 (15)
C5	0.048 (2)	0.0433 (17)	0.0387 (17)	−0.0096 (16)	−0.0154 (16)	−0.0070 (15)
C6	0.0338 (18)	0.0354 (15)	0.0388 (16)	−0.0109 (13)	−0.0118 (14)	−0.0005 (14)
C7	0.054 (2)	0.0309 (15)	0.049 (2)	−0.0136 (15)	−0.0180 (17)	0.0035 (15)
C8	0.057 (2)	0.0329 (16)	0.0454 (19)	−0.0040 (16)	−0.0121 (17)	−0.0037 (15)
C9	0.052 (2)	0.0379 (17)	0.0407 (18)	0.0017 (16)	−0.0082 (17)	−0.0048 (15)
C10	0.055 (2)	0.0391 (17)	0.0422 (18)	−0.0104 (17)	−0.0107 (17)	0.0027 (16)
C11	0.059 (3)	0.0356 (16)	0.0442 (19)	−0.0200 (17)	−0.0040 (17)	−0.0021 (15)
C12	0.046 (2)	0.0318 (16)	0.053 (2)	−0.0083 (15)	−0.0046 (17)	−0.0069 (16)
C13	0.042 (2)	0.0469 (19)	0.048 (2)	−0.0110 (16)	−0.0016 (17)	−0.0048 (17)
C14	0.064 (3)	0.058 (2)	0.048 (2)	−0.006 (2)	−0.017 (2)	0.0007 (19)
C15	0.058 (3)	0.049 (2)	0.0436 (19)	−0.0066 (18)	−0.0071 (18)	−0.0067 (17)
C16	0.053 (3)	0.080 (3)	0.055 (2)	−0.019 (2)	−0.022 (2)	−0.017 (2)
C17	0.048 (2)	0.076 (3)	0.052 (2)	−0.018 (2)	−0.0129 (19)	−0.022 (2)
C18	0.056 (3)	0.050 (2)	0.057 (2)	−0.0168 (19)	−0.019 (2)	−0.0073 (19)
C19	0.088 (3)	0.047 (2)	0.060 (2)	−0.021 (2)	−0.043 (2)	0.0001 (19)
C20	0.059 (3)	0.057 (2)	0.0376 (17)	−0.0276 (19)	−0.0206 (17)	0.0026 (17)
C21	0.079 (3)	0.100 (3)	0.041 (2)	−0.059 (3)	−0.018 (2)	0.011 (2)
C22	0.065 (3)	0.144 (5)	0.043 (2)	−0.049 (4)	−0.009 (2)	−0.001 (3)
C23	0.081 (4)	0.110 (4)	0.047 (2)	−0.012 (3)	−0.015 (2)	−0.030 (3)
C24	0.073 (3)	0.060 (2)	0.051 (2)	−0.020 (2)	−0.017 (2)	−0.013 (2)
C25	0.048 (2)	0.0498 (19)	0.0366 (16)	−0.0219 (17)	−0.0159 (16)	0.0032 (16)
C26	0.051 (2)	0.064 (2)	0.0449 (19)	−0.026 (2)	−0.0159 (18)	0.0026 (18)
C27	0.075 (3)	0.061 (2)	0.049 (2)	−0.020 (2)	−0.017 (2)	0.004 (2)
C28	0.063 (3)	0.064 (3)	0.047 (2)	−0.020 (2)	−0.005 (2)	−0.001 (2)
C29	0.054 (3)	0.058 (2)	0.053 (2)	−0.020 (2)	−0.0076 (19)	−0.004 (2)
C30	0.056 (3)	0.056 (2)	0.055 (2)	−0.0096 (19)	−0.022 (2)	−0.0115 (19)
C31	0.051 (3)	0.057 (2)	0.053 (2)	−0.0085 (19)	−0.0183 (19)	−0.0155 (18)
C32	0.050 (3)	0.062 (2)	0.058 (2)	−0.017 (2)	−0.015 (2)	−0.013 (2)
C33	0.074 (3)	0.057 (2)	0.054 (2)	−0.021 (2)	−0.033 (2)	0.0028 (19)
C34	0.049 (2)	0.064 (2)	0.0389 (17)	−0.0257 (19)	−0.0177 (17)	0.0020 (17)
C35	0.064 (3)	0.086 (3)	0.047 (2)	−0.035 (3)	−0.016 (2)	0.002 (2)
C36	0.071 (4)	0.108 (4)	0.047 (2)	−0.024 (3)	−0.010 (2)	−0.013 (3)
C37	0.102 (5)	0.089 (4)	0.058 (3)	−0.020 (3)	−0.031 (3)	−0.025 (3)
C38	0.089 (4)	0.076 (3)	0.060 (3)	−0.042 (3)	−0.034 (3)	0.002 (2)
C39	0.060 (3)	0.069 (2)	0.0423 (19)	−0.031 (2)	−0.0258 (18)	0.0057 (19)
C40	0.054 (3)	0.096 (3)	0.051 (2)	−0.039 (2)	−0.017 (2)	0.006 (2)
N1	0.052 (2)	0.0457 (16)	0.0515 (17)	−0.0198 (15)	−0.0194 (15)	−0.0032 (14)
N2	0.062 (2)	0.060 (2)	0.0488 (18)	−0.0249 (18)	−0.0172 (17)	−0.0071 (16)
N3	0.070 (3)	0.0564 (19)	0.0496 (19)	−0.0232 (18)	−0.0090 (17)	−0.0071 (17)
N4	0.050 (2)	0.0619 (19)	0.0448 (17)	−0.0266 (17)	−0.0124 (15)	0.0048 (16)
N5	0.071 (3)	0.068 (2)	0.0485 (19)	0.001 (2)	−0.023 (2)	−0.0029 (19)
N6	0.100 (3)	0.0504 (19)	0.061 (2)	0.016 (2)	−0.036 (2)	−0.0224 (18)
N7	0.0403 (18)	0.0440 (15)	0.0406 (15)	−0.0167 (13)	−0.0046 (13)	0.0010 (13)
N8	0.058 (2)	0.0554 (18)	0.0439 (16)	−0.0223 (16)	−0.0093 (15)	−0.0044 (15)
N9	0.058 (2)	0.0583 (19)	0.0448 (17)	−0.0223 (17)	−0.0136 (16)	−0.0088 (15)
N10	0.055 (2)	0.0435 (15)	0.0468 (16)	−0.0206 (15)	−0.0211 (15)	−0.0006 (14)
O1	0.116 (3)	0.0512 (16)	0.099 (2)	−0.0303 (18)	−0.061 (2)	−0.0044 (17)
O2	0.059 (2)	0.0723 (19)	0.103 (3)	−0.0016 (17)	−0.033 (2)	−0.0103 (19)
O1W	0.067 (3)	0.118 (3)	0.067 (2)	−0.034 (2)	0.0094 (17)	−0.043 (2)
O3	0.098 (3)	0.0560 (16)	0.077 (2)	−0.0340 (17)	−0.046 (2)	0.0235 (16)
O2W	0.092 (3)	0.108 (3)	0.131 (4)	−0.050 (3)	−0.020 (3)	−0.010 (3)
O4	0.0386 (18)	0.113 (3)	0.0653 (18)	−0.0073 (17)	−0.0007 (14)	−0.0401 (18)
O3W	0.069 (3)	0.094 (3)	0.165 (5)	−0.008 (2)	−0.041 (3)	−0.005 (3)
O5	0.067 (2)	0.0644 (16)	0.0407 (13)	−0.0052 (14)	−0.0208 (13)	−0.0158 (12)
O4W	0.188 (6)	0.085 (3)	0.127 (4)	−0.043 (4)	−0.051 (4)	−0.010 (3)
O6	0.095 (2)	0.0394 (13)	0.0644 (17)	−0.0098 (14)	−0.0358 (17)	−0.0090 (13)
S1	0.0593 (7)	0.0375 (4)	0.0692 (6)	−0.0130 (4)	−0.0298 (5)	0.0011 (4)
S2	0.0410 (5)	0.0485 (5)	0.0396 (4)	−0.0005 (4)	−0.0116 (4)	−0.0141 (4)
Ag1	0.0758 (3)	0.0704 (2)	0.04346 (17)	−0.03161 (18)	−0.00942 (15)	−0.01273 (15)
Ag2	0.0871 (3)	0.0778 (2)	0.04749 (19)	−0.0295 (2)	−0.01030 (17)	−0.01494 (17)
Cl1	0.0453 (5)	0.0444 (4)	0.0475 (5)	−0.0044 (4)	−0.0209 (4)	−0.0021 (4)
Cl2	0.0497 (6)	0.0453 (5)	0.0618 (6)	0.0019 (4)	−0.0213 (5)	−0.0022 (4)
Cl3	0.0803 (8)	0.0638 (6)	0.0436 (5)	0.0058 (5)	−0.0142 (5)	−0.0120 (5)
Cl4	0.0791 (8)	0.0626 (6)	0.0467 (5)	−0.0158 (6)	0.0014 (5)	−0.0164 (5)

### Geometric parameters (Å, °)

**Table:**

C1—C2	1.382 (4)	C26—H26B	0.97
C1—C6	1.401 (4)	C27—C28	1.345 (6)
C1—S2	1.756 (3)	C27—N3	1.357 (6)
C2—C3	1.377 (5)	C27—H27	0.93
C2—H2	0.93	C28—N4	1.358 (6)
C3—C4	1.402 (5)	C28—H28	0.93
C3—Cl2	1.734 (3)	C29—N3	1.308 (5)
C4—N6	1.353 (5)	C29—N4	1.319 (5)
C4—C5	1.398 (5)	C29—H29	0.93
C5—C6	1.364 (5)	C30—C31	1.346 (6)
C5—H5	0.93	C30—N2	1.360 (6)
C6—Cl1	1.738 (3)	C30—H30	0.93
C7—C8	1.369 (5)	C31—N1	1.363 (5)
C7—C12	1.387 (5)	C31—H31	0.93
C7—S1	1.773 (4)	C32—N2	1.317 (5)
C8—C9	1.373 (5)	C32—N1	1.341 (5)
C8—Cl3	1.756 (4)	C32—H32	0.93
C9—C10	1.388 (5)	C33—N1	1.468 (5)
C9—H9	0.93	C33—C34^ii^	1.499 (6)
C10—N5	1.371 (5)	C33—H33A	0.97
C10—C11	1.383 (6)	C33—H33B	0.97
C11—C12	1.385 (5)	C34—C39	1.390 (5)
C11—Cl4	1.748 (4)	C34—C35	1.393 (6)
C12—H12	0.93	C34—C33^ii^	1.499 (6)
C13—N8	1.313 (5)	C35—C36	1.355 (7)
C13—N7	1.331 (5)	C35—H35	0.93
C13—H13	0.93	C36—C37	1.362 (8)
C14—C15	1.350 (6)	C36—H36	0.93
C14—N8	1.362 (6)	C37—C38	1.383 (8)
C14—H14	0.93	C37—H37	0.93
C15—N7	1.359 (5)	C38—C39	1.388 (7)
C15—H15	0.93	C38—H38	0.93
C16—C17	1.349 (6)	C39—C40	1.495 (6)
C16—N9	1.369 (6)	C40—N4	1.478 (5)
C16—H16	0.93	C40—H40A	0.97
C17—N10	1.370 (5)	C40—H40B	0.97
C17—H17	0.93	N2—Ag2	2.092 (3)
C18—N9	1.303 (5)	N3—Ag2	2.090 (4)
C18—N10	1.327 (5)	N5—H5A	0.84 (3)
C18—H18	0.93	N5—H5B	0.81 (6)
C19—N10	1.475 (4)	N6—H6A	0.83 (3)
C19—C20^i^	1.508 (6)	N6—H6B	0.80 (3)
C19—H19A	0.97	N8—Ag1	2.103 (3)
C19—H19B	0.97	N9—Ag1	2.100 (3)
C20—C21	1.387 (6)	O1—S1	1.430 (3)
C20—C25	1.394 (5)	O2—S1	1.443 (4)
C20—C19^i^	1.508 (6)	O1W—H1A	0.80 (6)
C21—C22	1.356 (8)	O1W—H1B	0.83 (7)
C21—H21	0.93	O3—S1	1.450 (3)
C22—C23	1.369 (8)	O2W—H2A	0.81 (6)
C22—H22	0.93	O2W—H2B	0.83 (3)
C23—C24	1.395 (7)	O4—S2	1.443 (3)
C23—H23	0.93	O3W—H3A	0.82 (7)
C24—C25	1.381 (6)	O3W—H3B	0.83 (3)
C24—H24	0.93	O5—S2	1.451 (3)
C25—C26	1.502 (5)	O4W—H4A	0.84 (10)
C26—N7	1.476 (4)	O4W—H4B	0.87 (8)
C26—H26A	0.97	O6—S2	1.449 (3)
			
C2—C1—C6	117.7 (3)	N3—C29—N4	111.7 (4)
C2—C1—S2	118.7 (2)	N3—C29—H29	124.1
C6—C1—S2	123.6 (2)	N4—C29—H29	124.1
C3—C2—C1	121.0 (3)	C31—C30—N2	110.0 (4)
C3—C2—H2	119.5	C31—C30—H30	125.0
C1—C2—H2	119.5	N2—C30—H30	125.0
C2—C3—C4	121.5 (3)	C30—C31—N1	105.9 (4)
C2—C3—Cl2	119.2 (3)	C30—C31—H31	127.1
C4—C3—Cl2	119.3 (3)	N1—C31—H31	127.1
N6—C4—C5	121.0 (3)	N2—C32—N1	110.6 (4)
N6—C4—C3	122.0 (3)	N2—C32—H32	124.7
C5—C4—C3	117.1 (3)	N1—C32—H32	124.7
C6—C5—C4	121.2 (3)	N1—C33—C34^ii^	112.0 (3)
C6—C5—H5	119.4	N1—C33—H33A	109.2
C4—C5—H5	119.4	C34^ii^—C33—H33A	109.2
C5—C6—C1	121.6 (3)	N1—C33—H33B	109.2
C5—C6—Cl1	117.6 (2)	C34^ii^—C33—H33B	109.2
C1—C6—Cl1	120.8 (3)	H33A—C33—H33B	107.9
C8—C7—C12	117.2 (3)	C39—C34—C35	118.6 (4)
C8—C7—S1	125.6 (3)	C39—C34—C33^ii^	122.9 (4)
C12—C7—S1	117.1 (3)	C35—C34—C33^ii^	118.5 (4)
C7—C8—C9	122.4 (4)	C36—C35—C34	121.9 (4)
C7—C8—Cl3	121.2 (3)	C36—C35—H35	119.1
C9—C8—Cl3	116.4 (3)	C34—C35—H35	119.1
C8—C9—C10	121.0 (4)	C35—C36—C37	119.5 (5)
C8—C9—H9	119.5	C35—C36—H36	120.2
C10—C9—H9	119.5	C37—C36—H36	120.2
N5—C10—C11	122.6 (4)	C36—C37—C38	120.5 (5)
N5—C10—C9	120.5 (4)	C36—C37—H37	119.7
C11—C10—C9	116.9 (3)	C38—C37—H37	119.7
C10—C11—C12	121.7 (4)	C37—C38—C39	120.3 (4)
C10—C11—Cl4	119.6 (3)	C37—C38—H38	119.8
C12—C11—Cl4	118.6 (3)	C39—C38—H38	119.8
C11—C12—C7	120.8 (4)	C38—C39—C34	119.1 (4)
C11—C12—H12	119.6	C38—C39—C40	118.3 (4)
C7—C12—H12	119.6	C34—C39—C40	122.6 (4)
N8—C13—N7	111.7 (3)	N4—C40—C39	112.6 (3)
N8—C13—H13	124.2	N4—C40—H40A	109.1
N7—C13—H13	124.2	C39—C40—H40A	109.1
C15—C14—N8	110.5 (4)	N4—C40—H40B	109.1
C15—C14—H14	124.8	C39—C40—H40B	109.1
N8—C14—H14	124.8	H40A—C40—H40B	107.8
C14—C15—N7	105.4 (4)	C32—N1—C31	107.6 (3)
C14—C15—H15	127.3	C32—N1—C33	125.1 (4)
N7—C15—H15	127.3	C31—N1—C33	127.3 (4)
C17—C16—N9	110.0 (4)	C32—N2—C30	105.9 (3)
C17—C16—H16	125.0	C32—N2—Ag2	128.8 (3)
N9—C16—H16	125.0	C30—N2—Ag2	125.3 (3)
C16—C17—N10	105.4 (4)	C29—N3—C27	104.9 (4)
C16—C17—H17	127.3	C29—N3—Ag2	124.5 (3)
N10—C17—H17	127.3	C27—N3—Ag2	130.6 (3)
N9—C18—N10	111.9 (4)	C29—N4—C28	107.8 (3)
N9—C18—H18	124.0	C29—N4—C40	125.9 (4)
N10—C18—H18	124.0	C28—N4—C40	126.2 (4)
N10—C19—C20^i^	112.7 (3)	C10—N5—H5A	112 (4)
N10—C19—H19A	109.1	C10—N5—H5B	113 (4)
C20^i^—C19—H19A	109.1	H5A—N5—H5B	126 (5)
N10—C19—H19B	109.1	C4—N6—H6A	124 (5)
C20^i^—C19—H19B	109.1	C4—N6—H6B	120 (5)
H19A—C19—H19B	107.8	H6A—N6—H6B	113 (6)
C21—C20—C25	118.5 (4)	C13—N7—C15	107.5 (3)
C21—C20—C19^i^	118.8 (4)	C13—N7—C26	126.3 (3)
C25—C20—C19^i^	122.7 (4)	C15—N7—C26	126.1 (4)
C22—C21—C20	121.5 (4)	C13—N8—C14	104.8 (3)
C22—C21—H21	119.3	C13—N8—Ag1	125.4 (3)
C20—C21—H21	119.3	C14—N8—Ag1	129.8 (3)
C21—C22—C23	120.5 (5)	C18—N9—C16	105.3 (3)
C21—C22—H22	119.7	C18—N9—Ag1	128.8 (3)
C23—C22—H22	119.7	C16—N9—Ag1	125.8 (3)
C22—C23—C24	119.5 (5)	C18—N10—C17	107.4 (3)
C22—C23—H23	120.3	C18—N10—C19	125.5 (4)
C24—C23—H23	120.3	C17—N10—C19	127.1 (4)
C25—C24—C23	120.2 (4)	H1A—O1W—H1B	104 (5)
C25—C24—H24	119.9	H2A—O2W—H2B	104 (6)
C23—C24—H24	119.9	H3A—O3W—H3B	105 (4)
C24—C25—C20	119.9 (4)	H4A—O4W—H4B	101 (4)
C24—C25—C26	118.0 (3)	O1—S1—O2	113.8 (2)
C20—C25—C26	122.1 (4)	O1—S1—O3	112.0 (2)
N7—C26—C25	112.3 (3)	O2—S1—O3	111.8 (2)
N7—C26—H26A	109.1	O1—S1—C7	108.49 (17)
C25—C26—H26A	109.1	O2—S1—C7	104.3 (2)
N7—C26—H26B	109.1	O3—S1—C7	105.73 (18)
C25—C26—H26B	109.1	O4—S2—O6	112.7 (2)
H26A—C26—H26B	107.9	O4—S2—O5	113.29 (19)
C28—C27—N3	110.4 (4)	O6—S2—O5	111.49 (18)
C28—C27—H27	124.8	O4—S2—C1	105.88 (18)
N3—C27—H27	124.8	O6—S2—C1	107.38 (17)
C27—C28—N4	105.2 (4)	O5—S2—C1	105.46 (16)
C27—C28—H28	127.4	N9—Ag1—N8	175.91 (13)
N4—C28—H28	127.4	N3—Ag2—N2	178.95 (15)
			
C6—C1—C2—C3	1.0 (5)	C33^ii^—C34—C39—C38	−179.2 (3)
S2—C1—C2—C3	−179.5 (3)	C35—C34—C39—C40	179.1 (4)
C1—C2—C3—C4	−1.5 (5)	C33^ii^—C34—C39—C40	0.5 (5)
C1—C2—C3—Cl2	179.6 (3)	C38—C39—C40—N4	−95.9 (4)
C2—C3—C4—N6	−179.4 (4)	C34—C39—C40—N4	84.4 (5)
Cl2—C3—C4—N6	−0.5 (6)	N2—C32—N1—C31	−0.5 (5)
C2—C3—C4—C5	0.9 (5)	N2—C32—N1—C33	−178.8 (3)
Cl2—C3—C4—C5	179.8 (3)	C30—C31—N1—C32	0.6 (5)
N6—C4—C5—C6	−179.6 (4)	C30—C31—N1—C33	178.9 (4)
C3—C4—C5—C6	0.1 (5)	C34^ii^—C33—N1—C32	−145.3 (4)
C4—C5—C6—C1	−0.6 (5)	C34^ii^—C33—N1—C31	36.7 (6)
C4—C5—C6—Cl1	178.6 (3)	N1—C32—N2—C30	0.2 (5)
C2—C1—C6—C5	0.0 (5)	N1—C32—N2—Ag2	−177.8 (2)
S2—C1—C6—C5	−179.4 (3)	C31—C30—N2—C32	0.2 (5)
C2—C1—C6—Cl1	−179.1 (2)	C31—C30—N2—Ag2	178.3 (3)
S2—C1—C6—Cl1	1.5 (4)	N4—C29—N3—C27	0.6 (5)
C12—C7—C8—C9	−1.1 (5)	N4—C29—N3—Ag2	178.8 (3)
S1—C7—C8—C9	−176.0 (3)	C28—C27—N3—C29	−0.6 (5)
C12—C7—C8—Cl3	179.4 (3)	C28—C27—N3—Ag2	−178.6 (3)
S1—C7—C8—Cl3	4.5 (5)	N3—C29—N4—C28	−0.4 (5)
C7—C8—C9—C10	1.6 (6)	N3—C29—N4—C40	−176.5 (3)
Cl3—C8—C9—C10	−178.9 (3)	C27—C28—N4—C29	0.0 (5)
C8—C9—C10—N5	176.3 (4)	C27—C28—N4—C40	176.1 (4)
C8—C9—C10—C11	−1.3 (6)	C39—C40—N4—C29	−135.6 (4)
N5—C10—C11—C12	−177.1 (4)	C39—C40—N4—C28	49.0 (6)
C9—C10—C11—C12	0.5 (5)	N8—C13—N7—C15	1.5 (4)
N5—C10—C11—Cl4	2.0 (5)	N8—C13—N7—C26	178.6 (3)
C9—C10—C11—Cl4	179.6 (3)	C14—C15—N7—C13	−1.2 (4)
C10—C11—C12—C7	0.0 (5)	C14—C15—N7—C26	−178.3 (4)
Cl4—C11—C12—C7	−179.1 (3)	C25—C26—N7—C13	124.6 (4)
C8—C7—C12—C11	0.3 (5)	C25—C26—N7—C15	−58.8 (5)
S1—C7—C12—C11	175.7 (3)	N7—C13—N8—C14	−1.2 (5)
N8—C14—C15—N7	0.5 (5)	N7—C13—N8—Ag1	179.5 (2)
N9—C16—C17—N10	0.1 (5)	C15—C14—N8—C13	0.4 (5)
C25—C20—C21—C22	−0.2 (6)	C15—C14—N8—Ag1	179.7 (3)
C19^i^—C20—C21—C22	−178.3 (4)	N10—C18—N9—C16	−0.5 (4)
C20—C21—C22—C23	0.0 (7)	N10—C18—N9—Ag1	175.9 (2)
C21—C22—C23—C24	0.6 (8)	C17—C16—N9—C18	0.2 (5)
C22—C23—C24—C25	−0.8 (7)	C17—C16—N9—Ag1	−176.3 (3)
C23—C24—C25—C20	0.6 (6)	N9—C18—N10—C17	0.6 (5)
C23—C24—C25—C26	179.5 (4)	N9—C18—N10—C19	178.9 (3)
C21—C20—C25—C24	−0.1 (5)	C16—C17—N10—C18	−0.4 (5)
C19^i^—C20—C25—C24	177.9 (3)	C16—C17—N10—C19	−178.7 (4)
C21—C20—C25—C26	−179.0 (3)	C20^i^—C19—N10—C18	157.7 (4)
C19^i^—C20—C25—C26	−1.0 (5)	C20^i^—C19—N10—C17	−24.3 (6)
C24—C25—C26—N7	104.5 (4)	C8—C7—S1—O1	−49.1 (4)
C20—C25—C26—N7	−76.6 (4)	C12—C7—S1—O1	135.9 (3)
N3—C27—C28—N4	0.4 (5)	C8—C7—S1—O2	−170.8 (3)
N2—C30—C31—N1	−0.5 (5)	C12—C7—S1—O2	14.2 (3)
C39—C34—C35—C36	−0.1 (6)	C8—C7—S1—O3	71.2 (4)
C33^ii^—C34—C35—C36	178.5 (4)	C12—C7—S1—O3	−103.8 (3)
C34—C35—C36—C37	1.0 (7)	C2—C1—S2—O4	115.0 (3)
C35—C36—C37—C38	−1.0 (8)	C6—C1—S2—O4	−65.6 (3)
C36—C37—C38—C39	0.3 (7)	C2—C1—S2—O6	−124.4 (3)
C37—C38—C39—C34	0.5 (6)	C6—C1—S2—O6	55.1 (3)
C37—C38—C39—C40	−179.2 (4)	C2—C1—S2—O5	−5.4 (3)
C35—C34—C39—C38	−0.6 (5)	C6—C1—S2—O5	174.1 (3)

Symmetry codes: (i) −*x*+1, −*y*+1, −*z*+2; (ii) −*x*+2, −*y*, −*z*+1.

### Hydrogen-bond geometry (Å, °)

**Table:**

*D*—H···*A*	*D*—H	H···*A*	*D*···*A*	*D*—H···*A*
O2W—H2A···O6	0.81 (6)	2.15 (7)	2.868 (5)	148 (7)
O3W—H3A···O2	0.82 (7)	2.00 (7)	2.819 (6)	172 (9)
O1W—H1A···O4^iii^	0.80 (6)	1.99 (6)	2.762 (5)	165 (7)
N6—H6B···O2^iv^	0.80 (3)	2.19 (4)	2.928 (5)	154 (6)
N5—H5B···O5^v^	0.81 (6)	2.28 (6)	2.913 (5)	136 (5)

Symmetry codes: (iii) *x*−1, *y*, *z*+1; (iv) *x*, *y*+1, *z*; (v) −*x*+1, −*y*+1, −*z*.

## References

[bb1] Aakeröy, C. B. & Beatty, A. M. (1998). *Chem. Commun.* pp. 1067–1068.

[bb2] Cote, A. P. & Shimizu, G. K. H. (2004). *Inorg. Chem.* **43**, 6663–6673. 10.1021/ic0491229 15476366

[bb3] Feazell, R. P., Carson, C. E. & Klausmeyer, K. (2006). *Inorg. Chem.* **45**, 2635–2643. 10.1021/ic051284x 16529486

[bb4] Higashi, T. (1995). *ABSCOR* Rigaku Corporation, Tokyo, Japan.

[bb5] Li, F.-F., Ma, J.-F., Song, S.-Y., Yang, J., Jia, H.-Q. & Hu, N.-H. (2006). *Cryst. Growth Des.* **6**, 209–215.

[bb6] Liu, H.-Y., Wu, H., Ma, J.-F., Song, S.-Y., Yang, J., Liu, Y.-Y. & Su, Z.-M. (2007). *Inorg. Chem.* **46**, 7299–7311. 10.1021/ic070147s 17685508

[bb7] Ma, J.-F., Yang, J., Li, S.-L., Song, S.-Y., Zhang, H.-J., Wang, H.-S. & Yang, K.-Y. (2005). *Cryst. Growth Des.* **5**, 807–812.

[bb8] Rigaku (1998). *PROCESS-AUTO* Rigaku Corporation, Tokyo, Japan.

[bb9] Sheldrick, G. M. (2008). *Acta Cryst.* A**64**, 112–122. 10.1107/S0108767307043930 18156677

